# Tricaine, eugenol and etomidate for repetitive procedural anesthesia in adult zebrafish, *Danio rerio*: effect on stress and behavior

**DOI:** 10.3389/fvets.2025.1562425

**Published:** 2025-05-14

**Authors:** Suneeta Narumanchi, Sanni Perttunen, Pyry Laine, Riikka Kosonen, Päivi Lakkisto, Mika Laine, Ilkka Tikkanen, Jere Paavola

**Affiliations:** ^1^Unit of Cardiovascular Research, Minerva Foundation Institute for Medical Research, Biomedicum Helsinki, Helsinki, Finland; ^2^Clinical Chemistry and Hematology, Helsinki University and Helsinki University Hospital, Helsinki, Finland; ^3^Heart and Lung Center, Helsinki University and Helsinki University Hospital, Helsinki, Finland; ^4^Abdominal Center, Nephrology, Helsinki University and Helsinki University Hospital, Helsinki, Finland

**Keywords:** zebrafish, health and welfare, procedure, anesthesia, stress

## Abstract

Zebrafish has emerged as a popular animal model in biomedical research. Numerous procedures and interventions require occasionally repetitive anesthesia. Tricaine is the most frequently used anesthetic for zebrafish and its efficacy is well established. However, the safety and efficacy of other anesthetics used for zebrafish require further examination, especially regarding repetitive anesthesia. Hence, we compare three anesthetics: tricaine (150 mg/l), eugenol (55 mg/l) and etomidate (4 mg/l) in wildtype adult zebrafish with and without interventions in the form of intraperitoneal injections. Groups of fish receiving the injections are named as (+ injection). We quantify anesthesia induction and recovery times as well as swimming behavior and cortisol levels as indicators of stress. Swimming behavior is quantified with the novel tank method as tank preference and number of turnings. Adult zebrafish are randomly divided into seven groups; tricaine (*n* = 15), tricaine (+injection; *n* = 15), eugenol (*n* = 15), eugenol (+injection; *n* = 14), etomidate (*n* = 15), etomidate (+injection; *n* = 15) and sham (*n* = 10), and anesthetized until they reach stage 4 anesthesia, daily for 10 days. Following anesthesia induction, injection groups are given daily intraperitoneal injections with 0.9% saline (4 ml/kg) before transfer to a recovery tank to study the effect of a painful procedure (the intraperitoneal injection) during anesthesia on stress. The novel tank method is used for analyzing behavior at day 2 (beginning), day 5/6 (middle) and day 10 (end). Chronic stress is evaluated by whole-body cortisol measurement at the end of the 10-day experiment. Additionally, acute stress is evaluated by whole-body cortisol measurement 30 min after single anesthesia in five groups: tricaine (*n* = 5), eugenol (*n* = 5), etomidate (*n* = 5), sham (*n* = 5), and untreated controls (*n* = 5). We find that fish anesthetized with tricaine recover fast (~ 1.5 min) and show normal swimming behavior. Fish anesthetized with eugenol show recovery time (~ 2.5 min) and swimming behavior similar to that of fish receiving tricaine. Fish anesthetized with etomidate have the longest recovery time (~ 5.5 min) and exhibit stressed swimming behavior. Cortisol levels remain at similar levels. Our study supports the use of tricaine as the anesthetic-of-choice for repetitive anesthesia of short duration in zebrafish, followed by eugenol.

## 1 Introduction

Zebrafish (*Danio rerio*) has in recent decades become a popular vertebrate model in biomedical research for studying the genetic basis as well as molecular and cellular mechanisms of human diseases and investigating the toxicology and therapeutic potential of small molecules (1, 2). These studies often include the need for anesthesia.

An ideal anesthetic is non-toxic to subject and handler, induces deep anesthesia without impairing vital organ function or causing involuntary movements, and permits fast recovery of the subject (3). The mechanisms of action for inducing anesthesia are known for routinely used anesthetics, whereas the effect of repetitive anesthesia on stress in zebrafish remains incompletely understood. Tricaine is the most used anesthetic agent for zebrafish (4). It enters the body via the gills and produces anesthesia by impeding neuronal signal transmission peripherally to the central nervous system. During tricaine anesthesia hyperglycemia (5), increased heart rate and increased respiration rate are observed, followed by depression of heart rate and ventilation (6, 7). Eugenol has been widely reported to be safe and effective for anesthesia in zebrafish (8). It has been used to anesthetize zebrafish e.g., for caudal fin clipping (9) and facial injection of acetic acid (10). However, eugenol is reported to have bigger effects on respiratory and cardiac systems than tricaine, resulting in lower ventilation and heart rates (11). Furthermore, toxicity of eugenol has been reported (12). Metomidate is a hypnotic and used for anesthetizing fish during transportation to reduce stress (5). However, metomidate is not suitable for surgical procedures as it fails to provide analgesia or sufficient anesthesia for surgical procedures (13). Etomidate, which is a derivative of metomidate, causes a slight drop in blood pressure and muscle twitching (14–16), and has been found to be suboptimal for anesthesia also in zebrafish (17). On the other hand, zebrafish seem to find tricaine more aversive than eugenol or metomidate, suggesting the latter two might be better options than tricaine for zebrafish euthanasia (18). Additionally, tricaine inhibits neural voltage-gated sodium channels, raising the possibility it might not be the optimal anesthetic agent for use in neuroscience (19).

Many experimental treatments and procedures require repetitive anesthesia, which raises additional challenges. Repetitive anesthesia with tricaine has been shown to increase mortality and aversiveness, while providing insufficient analgesia post-operatively (20).

Stress is known to activate several endocrine pathways in humans and animals for responding to the challenge. One of these is the hypothalamic-pituitary-interrenal axis. The hypothalamus produces corticotropin releasing factor, which stimulates release of adrenocorticotropic hormone from the anterior pituitary gland. In the presence of adrenocorticotropic hormone, the interrenal tissue synthesizes glucocorticoid hormone, mediating the stress response. In zebrafish cortisol is produced in response to stress. Whole-body cortisol techniques have been used in toxicological procedures (21) and for investigating crowding stress (22, 23). By modulating the stress response, the hypothalamic-pituitary-interrenal axis causes changes in fish behavior.

The novel tank method is commonly used for behavioral studies with zebrafish (24–26). Moving to a new, unfamiliar tank leads to anxiety and the fish spend more time diving and hiding at the bottom of the tank. Time spent in the top-part of the tank (tank preference) is measured to evaluate stress (26–29). Stress is known to affect fish movement (30, 31). Movement can be measured by turning, quantified as the number of times a fish turns and swims well. Decreased movement may be due to freezing (e.g., by high stress) or immobility (e.g., by sedative agents). Here decreased turning after recovery from anesthesia is interpreted as increased stress/prolonged recovery. Analysis of both endocrine and behavioral studies allows robust evaluation of stress caused by anesthetics.

By administering three different anesthetics, we sought to evaluate the effect of repetitive anesthesia on anesthesia induction and recovery times, post-anesthesia swimming behavior, and cortisol levels in adult zebrafish. By comparing tricaine, eugenol, and etomidate we aimed to evaluate whether the use of tricaine as the preferred anesthetic in experimental zebrafish research can be challenged in terms of reduced stress and improved recovery after anesthesia manifested by reduced cortisol levels and improved/normalized swimming behavior, during a 10-day experimental protocol with daily anesthesia.

## 2 Materials and methods

### 2.1 Animals and housing

One **hundred** and **twenty-five** wildtype Turku mixed-sex adult zebrafish aged 12–18 months (32) maintained according to Westerfield (33) at the Biomedicum zebrafish unit (University of Helsinki, Finland) were used, as this age and type of zebrafish are commonly used in biomedical research. The fish were housed in 10-liter (l) tanks, at a density of 1.5 fish/l (15 fish/10-liter tank) on a recirculating system (Aquatic Habitats) in 28.5°C water (conductivity, 400 to 600 μS/cm; pH, 7.2 to 7.6; dissolved oxygen, >6 mg/l; ammonia, 0 ppm; nitrate, 0 to 0.5 ppm; and nitrite, 0 ppm) in a room with a 14:10 h light:dark cycle. System water was aerated and active carbon-filtered municipal tap water, filtered through a 20 μm particulate filter, and exposed to 80 W UV-light. The fish were fed **three** times daily with both a commercially pelleted diet (Special Diet Services, SDS400) and Artemia nauplii (Sanders, Great Salt Lake Artemia). The permit for zebrafish experiments was obtained from the Regional State Administrative Agency for Southern Finland in agreement with the ethical guidelines of the European convention (ESAVI/2988/04.10.07/2014, ESAVI/4131/04.10.07/2017 and ESAVI/16286/2020).

### 2.2 Anesthetic solutions

The anesthetics used were tricaine (Tricaine methanesulfonate or MS-222; E10521-106-10g; Sigma–Aldrich, St. Louis, MO, USA), eugenol (46129-100 ml-F; Sigma–Aldrich, St. Louis, MO, USA) and etomidate (E6530-10mg; Sigma–Aldrich, St. Louis, MO, USA). Stock solutions: tricaine 4 g/l buffered to pH 7 using Tris buffer pH 9 and stored in –20°C, eugenol 100 g/l in ethanol and stored at room temperature (RT) protected from light, etomidate 1 g/l in ethanol and stored at RT protected from light. Working solutions were made in fish water on each day of the experiment. Concentrations for working solutions were chosen from literature; tricaine 150 mg/l, eugenol 55 mg/l, etomidate 4 mg/l (16, 34, 35). On the last day of the experiment, the fish were euthanized with a lethal dose of the individual anesthetic used (three times as high as working concentrations); tricaine 450 mg/l, eugenol 165 mg/l, etomidate 12 mg/l.

### 2.3 Interventions

Interventions and behavioral assays were performed under the same environmental conditions as described for husbandry. A total number of 125 fish were used in this study. Groups of fish receiving the intraperitoneal injections are named as (+ injection). Fish for the experiments were selected randomly by an experienced technician in the fish lab who was unaware of the details of this project. Fish were randomly divided into the following groups (10-liter tank/group) for the 10-day experimental protocol: tricaine (*n* = 15), tricaine (+injection; *n* = 15), eugenol (*n* = 15), eugenol (+injection; *n* = 14), etomidate (*n* = 15), etomidate (+injection; *n* = 15), and sham (*n* = 10). Additionally, 25 fish were used for measuring acute stress in **five** groups: tricaine (*n* = 5), eugenol (*n* = 5), etomidate (*n* = 5), sham (*n* = 5), and untreated controls (*n* = 5). Fish were transferred with a net, weighed, and kept in the anesthetic solution until they reached stage 4 anesthesia (equilibrium and muscle tone are lost, and opercular movement decreases. No movement in response to touch). The fish were then transferred into a novel tank gently with a plastic spoon. Fish in the anesthetic (+injection) groups were transferred from the anesthetic solution onto a wet sponge and given intraperitoneal injections with 0.9% saline (4 ml/kg) before transfer to a novel tank, thus prolonging the transfer time between anesthesia induction and transfer to the novel tank to approximately **1** min in the (+injection) groups. This was repeated daily for 10 days for the seven groups of fish. All injections were given by a single researcher, and the depth of anesthesia prior to injection was tested by squeezing the tail with tweezers. **Ten** fish in the sham group were handled without anesthetics. These sham fish were moved **one** by **one** to a small beaker for “anesthesia” for **1** min and then moved to a novel tank. A 2-liter tank filled with ~2 l water (21 x 11 x 9 cm^3^) was used as the novel tank. Five-minute videos were recorded always at noon ±2 h with an iPad (Apple Inc., Cupertino, California, USA) at a distance of 15 cm from the long edge of the novel tank, which was 21 cm long, 11 cm wide and had a water depth of 9 cm. Videos were recorded at days 2, 5, and 10 for the tricaine, etomidate, and sham groups, and at days 2, 6, and 10 for the eugenol groups. All fish in a group remained in the same tank during the video recordings to mimic the real situation when anesthetizing fish for experiments. This procedure was chosen to maintain the daily experimental routine for the fish as invariable as possible on days with and without video recordings. Following video recordings, the fish were transferred to their group's respective tank with a net. All videos were observed and analyzed by a single observer. On the last day of the experiment, fish receiving anesthetics were euthanized with a lethal dose of the individual anesthetic used (tricaine 450 mg/l, eugenol 165 mg/l, etomidate 12 mg/l), in the afternoon. The sham fish and the untreated control fish were euthanized with ice. For acute stress measurements, additional fish not part of the 10-day experimental protocol were anesthetized, after recovery allowed to swim for 30 min in the novel tank and then euthanized with a lethal dose of the respective anesthetic or ice. All fish were weighed individually, snap-frozen in liquid nitrogen and stored at –80°C until cortisol extraction. Euthanizing and freezing for all groups were done at the same time of the day to avoid variation in cortisol levels due to differences in the time of day. Fish weight was monitored (Mettler Toledo PB 3002-S/PH Delta Range, Greifensee, Switzerland) daily as a part of the anesthetization process. The final weighing was performed with a more precise scale (Mettler Toledo XS 205 Dual Range, Greifensee, Switzerland).

### 2.4 Anesthesia induction time and recovery time

The fish were anesthetized until they reached stage 4 anesthesia, and the induction time was recorded (35). During anesthesia, immobility and reduced opercular movements were observed before taking the fish to experiments. These are external signs of loss of consciousness during stage 4 anesthesia (36–39). The recovery time was recorded from transfer to the novel tank until the fish could maintain a stable swimming position. Anesthesia induction and recovery times were recorded on days 2, 5 and 10.

### 2.5 Tank preference and turning

Time spent in the top-part of the novel tank was used to evaluate stress. The water column in the novel tank was divided into **two** halves with a thin horizontal tape on the side of the tank: top-part and bottom-part. Swimming behavior was recorded for 5 min after recovery from anesthesia. After anesthesia (+injection), videos were recorded at days 2, 5/6, and 10. The time spent at the top-part of the novel tank and turning was evaluated from recorded videos. The fish was considered to enter the top-part/bottom-part of the tank when its whole body had crossed the midline of the novel tank. For the turning, only those turnings were counted where the fish turned 180°. The total amount of turnings in 5 min was counted.

### 2.6 Cortisol measurement

Cortisol levels were measured to determine stress levels of individual fish (3, 24, 31). Chronic stress was evaluated following the 10-day experimental protocol. Additionally, cortisol measurement was used to evaluate acute stress in **five** groups of fish: tricaine, eugenol, etomidate, sham and control groups. Briefly, fish were anesthetized, after recovery from anesthesia allowed to swim for 30 min, and then euthanized with the respective anesthetic or ice. Control fish were euthanized with ice, otherwise unhandled. Fish were frozen in liquid nitrogen and stored in –80°C until samples were prepared. Sample preparation was modified from Canavello et al. (40). Each fish was processed as a single sample and weighed. Briefly, fish were partially thawed on ice and cut. **Four** times weight/volume of ice-cold PBS was added, and the sample was homogenized with Tissue-Tearor (Biospec products, Bartlesville, OK, USA). Homogenates were stored in –80°C until cortisol was extracted. Later, cortisol was extracted from 250 μl of homogenate. When 250 μl of homogenate was not available, the volume was decreased and the change in volume was considered in calculations. Ten-fold volume of diethyl ether was added to the homogenate and centrifuged at 3,500 rpm for 15 min at +4°C, followed by freezing at –20°C overnight. The ether phase was collected and evaporated in the fume hood at RT for 3.5 h (40). Samples were reconstituted in 250 μl enzyme immunoassay buffer. High concentration samples were diluted 1:2 and cortisol was measured according to the Cortisol ELISA kit (Cayman Chemical Company, Michigan, USA).

### 2.7 Statistical analysis

Data was analyzed with non-parametric Kruskall-Wallis test followed by Dunn's test with Prism software (GraphPad Prism 10, Dotmatics, Boston, MA, USA). Error bars represent standard error of mean (SEM) ^*^ *P* ≤ 0.05, ^**^ *P* ≤ 0.01, ^***^ *P* ≤ 0.001.

## 3 Results

### 3.1 Weight and mortality

The weight of each fish was measured daily from day 1 to day 10, and no weight change is observed during this time (Table 1). Mortality is highest in the eugenol fish that receive injections (33%). The etomidate fish, as well as the etomidate and the tricaine fish that received injections, have similar mortality (7%), whereas there is no mortality in the sham, the tricaine and the eugenol groups (Table 1). Only one fish in the etomidate group died in fish that did not receive injections.

**Table 1:** Weight and mortality.

**Group**	**Weight (g;** ±**SEM)**						**Mortality**	
	**1. day**	***n***	**5. day**	***n***	**10. day**	***n***	***n***	**%**
Sham	0.36 (0.03)	10	0.31 (0.02)	10	0.36 (0.02)	10	0	0
Tricaine	0.64 (0.04)	15	0.61 (0.04)	15	0.59 (0.03)	15	0	0
Tricaine +i.p. inj.	0.52 (0.03)	15	0.52 (0.03)	15	0.49 (0.03)	14	1	7
Eugenol	0.53 (0.03)	15	0.51 (0.03)	15	0.49 (0.03)	15	0	0
Eugenol +i.p. inj.	0.50 (0.03)	15	0.50 (0.03)	13	0.48 (0.03)	10	5	33
Etomidate	0.51 (0.03)	15	0.48 (0.03)	15	0.53 (0.03)	14	1	7
Etomidate +i.p. inj.	0.51 (0.03)	15	0.49 (0.03)	15	0.48 (0.03)	14	1	7

### 3.2 Anesthesia induction time

**Figure 1:**
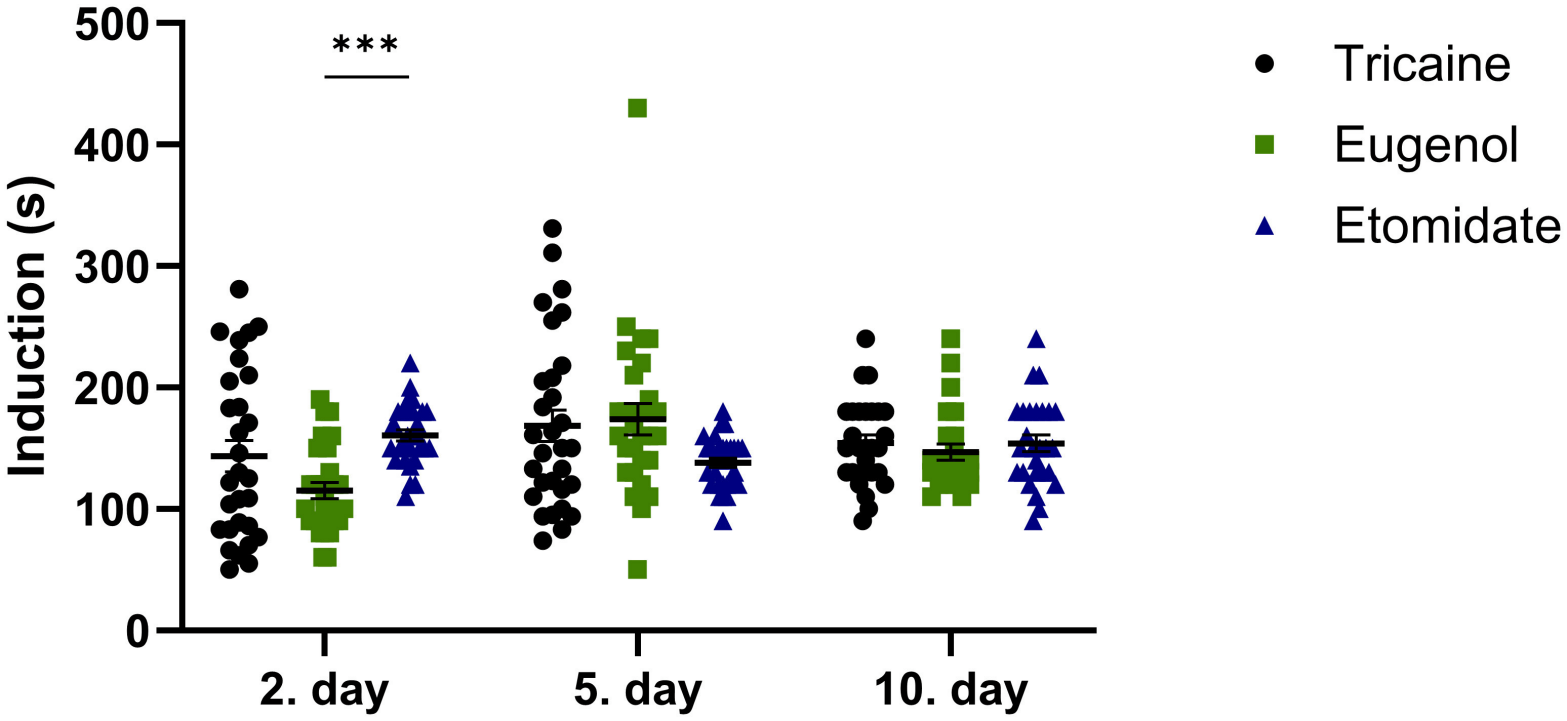
Anesthesia induction time (seconds, s). Tricaine 2. day *n* = 29, 6. day *n* = 30, 10. day *n* = 30. Eugenol 2. day *n* = 29, 5. day *n* = 28, 10. day *n* = 25. Etomidate 2. day *n* = 29, 5. day *n* = 30, 10. day *n* = 28. Error bars represent standard error of mean (SEM), ***P ≤ 0.001.

The anesthesia induction time at day 2 is shorter for eugenol compared to etomidate (*P* = 0.0007; Figure 1). At other timepoints, all anesthetics show similar anesthesia induction times.

### 3.3 Anesthesia recovery time

**Figure 2:**
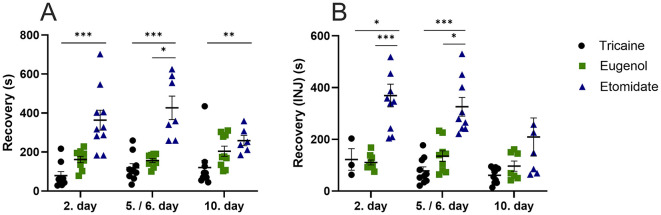
Anesthesia recovery time (seconds, s) **(A)**, with injections **(B)**. **(A)** Tricaine 2. day *n* = 9, 5. day *n* = 10, 10. day *n* = 10. Eugenol 2. day *n* = 9, 6. day *n* = 9, 10. day *n* = 10. Etomidate 2. day *n* = 10, 5. day *n* = 7, day *n* = 6. **(B)** Tricaine 2. day *n* = 3, 5. day *n* = 10, 10. day *n* = 10. Eugenol 2. day *n* = 9, 6. day *n* = 9, 10. day *n* = 7. Etomidate 2. day *n* = 10, 5. day *n* = 9, 10. day *n* = 7. Error bars represent standard error of mean (SEM), *P ≤ 0.05, **P ≤ 0.01, ***P ≤ 0.001.

The recovery time from anesthesia is longer for etomidate compared to tricaine at day 2 (*P* = 0.0001), day 5 (*P* = 0.0002) and day 10 (*P* = 0.0067; Figure 2A). In fish that received injections, anesthesia recovery time is longer for etomidate compared to tricaine at day 2 (*P* = 0.03) and day 5 (*P* = 0.0001; Figure 2B). Recovery time is also longer for etomidate compared to eugenol at day 5 (*P* = 0.02; Figure 2A). In fish that received injections, recovery after anesthesia is longer for etomidate compared to eugenol at day 2 (*P* = 0.0006) and day 5 (*P* = 0.02; Figure 2B). The recovery time is similar in all fish that received injections at day 10 (Figure 2). Tricaine and eugenol show similar recovery times.

### 3.4 Swimming behavior in the novel tank

**Figure 3:**
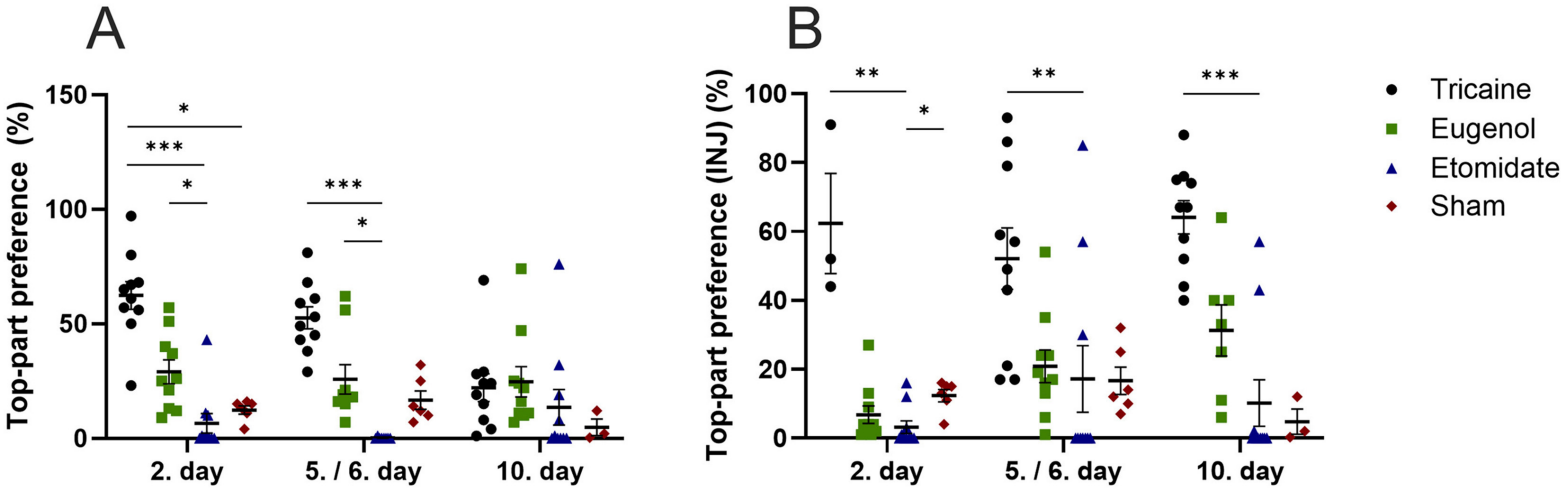
Top-part preference in the novel tank (%) **(A)**, with injections **(B)**. **(A)** Tricaine 2. day *n* = 10, 5. day *n* = 10, 10. day *n* = 10. Eugenol 2. Day *n* = 10, 6. day *n* = 9, 10. day *n* = 10. Etomidate 2. day *n* = 10, 5. day *n* = 8, 10. day *n* = 10. Sham 2. day *n* = 6, 5. day *n* = 6, 10. day *n* = 3. **(B)** Tricaine 2. day *n* = 3, 5. day *n* = 10, 10. day *n* = 10. Eugenol 2. day *n* = 10, 6. day *n* = 10, 10. day *n* = 7. Etomidate 2. day *n* = 10, 5. day *n* = 10, 10. day 10. *n* = 10. Sham 2. day *n* = 6, 5. day *n* = 6, 10. day *n* = 3. Error bars represent standard error of mean (SEM) *P ≤ 0.05, **P ≤ 0.01, ***P ≤ 0.001.

**Figure 4:**
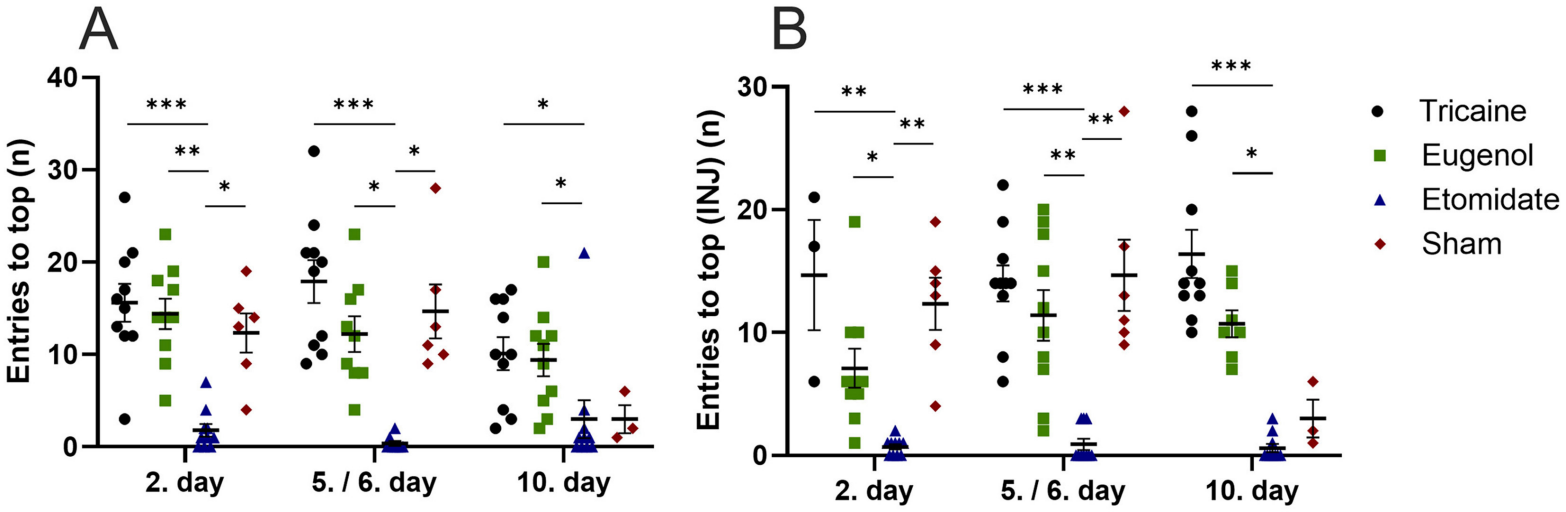
Entries to the top-part of the novel tank (number, n) **(A)**, with injections **(B)**. **(A)** Tricaine 2. day *n* = 10, 5. day *n* = 10, 10. day *n* = 10. Eugenol 2. Day *n* = 10, 6. day *n* = 9, 10. day *n* = 10. Etomidate 2. day *n* = 10, 5. day *n* = 8, 10. day *n* = 10. Sham 2. day *n* = 6, 5. day *n* = 6, 10. day *n* = 3. **(B)** Tricaine 2. day *n* = 3, 5. day *n* = 10, 10. day *n* = 10. Eugenol 2. day *n* = 10, 6. day *n* = 10, 10. day *n* = 7. Etomidate 2. day *n* = 10, 5. day *n* = 10, 10. day 10. *n* = 10. Sham 2. day *n* = 6, 5. day *n* = 6, 10. day *n* = 3. Error bars represent standard error of mean (SEM) *P ≤ 0.05, **P ≤ 0.01, ***P ≤ 0.001.

**Figure 5:**
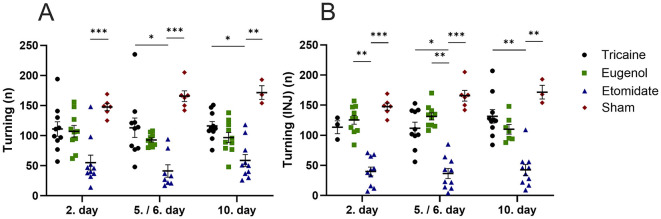
Turning in the novel tank (number, n) **(A)**, with injections **(B)**. **(A)** Tricaine 2. day *n* = 10, 5. day *n* = 10, 10. day *n* = 10. Eugenol 2. Day *n* = 10, 6. day *n* = 9, 10. day *n* = 10. Etomidate 2. day *n* = 10, 5. day *n* = 8, 10. day *n* = 10. Sham 2. day *n* = 6, 5. day *n* = 6, 10. day *n* = 3. **(B)** Tricaine 2. day *n* = 3, 5. day *n* = 10, 10. day *n* = 10. Eugenol 2. day *n* = 10, 6. day *n* = 10, 10. day *n* = 7. Etomidate 2. day *n* = 10, 5. day *n* = 10, 10. day 10. *n* = 10. Sham 2. day *n* = 6, 5. day *n* = 6, 10. day *n* = 3. Error bars represent standard error of mean (SEM) *P ≤ 0.05, **P ≤ 0.01, ***P ≤ 0.001.

Tricaine fish show more top-part preference in the novel tank compared to etomidate fish at day 2 (*P* = 0.0001) and day 5 (*P* = 0.0001) as well as compared to sham fish (*P* = 0.02) at day 2. Eugenol fish also show more top-part preference in the novel tank compared to etomidate fish at day 2 (*P* = 0.04) and day 5 (*P* = 0.02). All fish spend similar times in top-part of novel tank on day 10 (Figure 3A). In fish that received injections, tricaine fish show more top-part preference compared to etomidate fish at day 2 (*P* = 0.003), day 5 (*P* = 0.004) and day 10 (*P* = 0.0001). In addition, sham fish show more top-part preference compared to etomidate fish at day 2 (*P* = 0.04; Figure 3B). Tricaine fish show more entries to the top-part of the novel tank compared to etomidate fish at all time-points (day 2 *P* = 0.0004; day 5 *P* = 0.0001 and day 10 *P* = 0.01). Eugenol fish also show more entries to top-part of the novel tank compared to etomidate fish at all time-points (day 2 *P* = 0.001; day 5 *P* = 0.02 and day 10 *P* = 0.02). Sham fish show more entries to top-part of the novel tank compared to etomidate fish at day 2 (*P* = 0.04) and day 5 (*P* = 0.01; Figure 4A). In fish that received injections, both tricaine fish (day 2 *P* = 0.006; day 5 *P* = 0.0003 and day 10 *P* = 0.0001) and eugenol fish (day 2 *P* = 0.02; day 5 *P* = 0.006 and day 10 *P* = 0.01) show more entries to top-part of novel tank compared to etomidate fish. Sham fish show more entries to top-part of novel tank compared to etomidate fish at day 2 (*P* = 0.001) and day 5 (*P* = 0.005; Figure 4B). Therefore, etomidate fish spend the least time in the top-part of the novel tank and enter it the least often. Sham fish show more turning compared to etomidate fish at day 2 (*P* = 0.0003), day 5 (*P* = 0.0001) and day 10 (*P* = 0.001). Tricaine fish show more turning compared to etomidate fish at day 5 (*P* = 0.03) and day 10 (*P* = 0.01; Figure 5A). In fish that received injections, sham fish show the most turning compared to etomidate fish at day 2 (*P* = 0.0001), day 5 (*P* = 0.0001) and day 10 (*P* = 0.002). Additionally, tricaine fish (day 5 *P* = 0.04 and day 10 *P* = 0.001) and eugenol fish (day 2 *P* = 0.003 and day 5 *P* = 0.002) show more turning compared to etomidate fish (Figure 5B).

### 3.5 Cortisol levels

**Figure 6:**
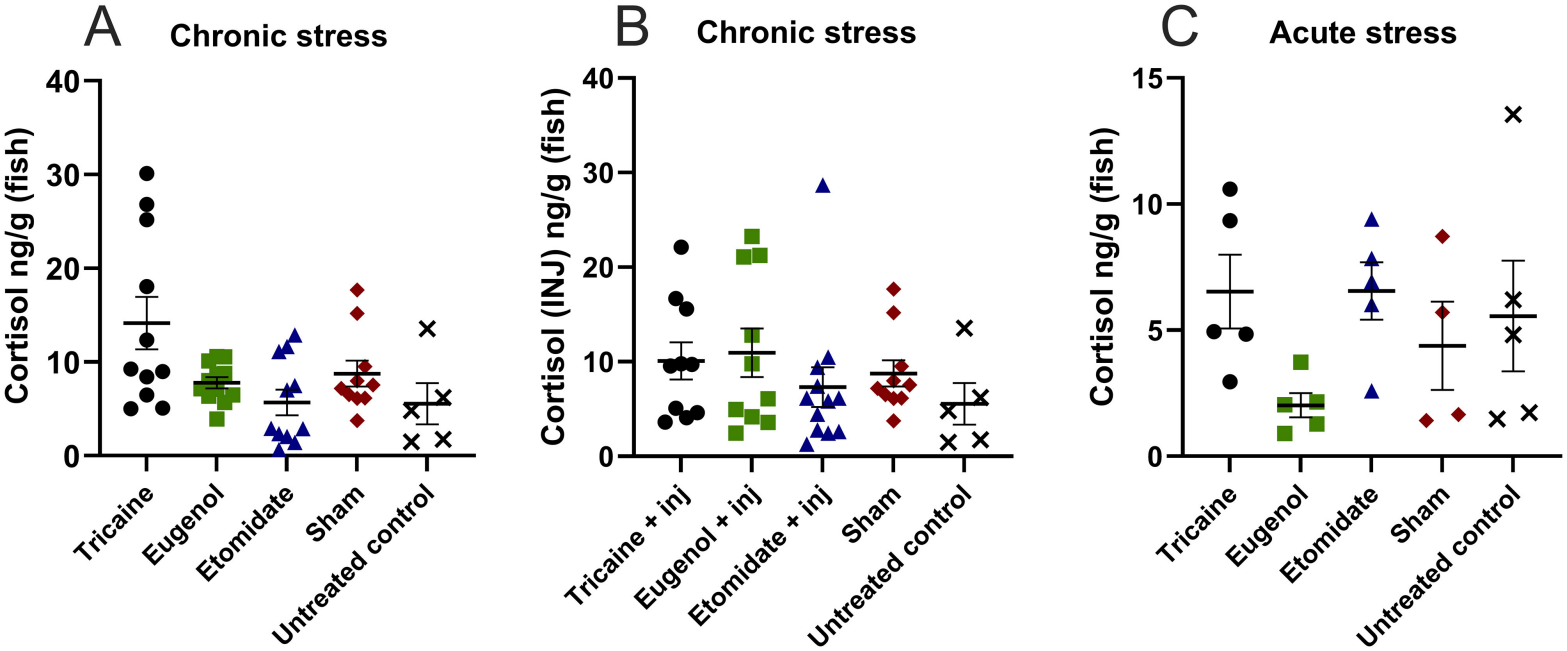
Cortisol levels (ng/g/fish) show no statistical difference. Chronic stress after the 10-day experimental protocol **(A)**, chronic stress after the 10-day experimental protocol in fish that received injections (INJ) **(B)**. Acute stress after a single anesthetic treatment **(C)**. **(A)** Tricaine *n* = 11. Eugenol *n* = 12. Etomidate *n* = 11. Sham *n* = 10. Untreated control *n* = 5. **(B)** Tricaine *n* = 11. Eugenol *n* = 10. Etomidate *n* = 12. Sham *n* = 10. Untreated control *n* = 5. **(C)** Tricaine *n* = 5. Eugenol *n* = 5. Etomidate *n* = 5. Sham *n* = 5. Untreated control *n* = 5. Error bars represent standard error of mean (SEM).

Chronic stress evaluated after the 10-day protocol is similar in all groups, including sham and untreated controls (Figure 6A). Additionally, cortisol levels remain similar in fish that received injections (Figure 6B). Acute stress evaluated after a single anesthetic treatment is similar in all groups, including sham and untreated controls (Figure 6C). Furthermore, for each anesthetic the cortisol levels are similar with and without injections, and chronically vs. acutely.

## 4 Discussion

The aim of this study is to determine the most suitable anesthetic for zebrafish experiments containing procedures that require repetitive anesthesia for short duration. Tricaine, eugenol and etomidate are compared. Our study reveals that fish anesthetized with tricaine recover fast and show normal swimming behavior and stress. Fish anesthetized with etomidate have the longest recovery time and exhibit stressed swimming behavior. Fish anesthetized with eugenol show swimming behavior similar to fish receiving tricaine. Cortisol levels remain at similar levels. Therefore, our study supports the use of tricaine for repetitive anesthesia of short duration in zebrafish, followed by eugenol.

Thus far, tricaine is clearly the most researched of these anesthetic agents (41). Our results are in agreement with a recent comparison by Ayala-Soldado et al. between tricaine and eugenol for repetitive anesthesia (once daily for 3 days, no interventions) in adult zebrafish, taking into account that Ayala-Soldado et al. used a higher dose of eugenol compared to our study (85 mg/l vs. 55 mg/l) (42). The observed anesthesia induction time with eugenol was faster, and the anesthesia recovery time was correspondingly longer and prolonged during the 3-day study protocol. The exposure time to the anesthetic was 10 min, whereas we rapidly transferred the fish to recover after anesthesia had been induced, possibly explaining the longer anesthesia recovery times seen in that study. The authors discussed that the progressively prolonged anesthesia recovery time with eugenol may be due to its accumulation due to lipophilicity. Our results partly support this; although we did not observe significant prolongation of the anesthesia recovery time, we found that while there is no statistically significant difference, there is a trend of increased cumulative stress when comparing cortisol levels in eugenol fish acutely (9 ng/g) to chronic stress after the 10-day experimental protocol (29 ng/g). Musk et al. compared isoeugenol, which is structurally similar to eugenol and also a constituent of clove oil, to tricaine for use in anesthesia for caudal fin clipping and found their safety and efficacy to be similar (9). Ayala-Soldado et al. also compared eugenol and tricaine for zebrafish euthanasia using high doses of these anesthetics and found eugenol to be very potent and cause branchial alterations (43).

The weight of zebrafish remained unchanged during the 10-day experimental protocol (Table 1). For clarity, the weight of the fish used for acute stress measurements is shown in Supplementary Table 1. Eugenol fish that received injections had the highest mortality rate (33%). The etomidate fish, as well as the etomidate and the tricaine fish that received injections, had similar mortality (7%), whereas there was no mortality in the sham, tricaine and eugenol groups (Table 1). High mortality in the eugenol fish that received injections is in contrast with previous reports on eugenol's safety (34, 44). However, the higher mortality is likely due to complications from the i.p. injections, as eugenol fish without injections showed no mortality. These results are consistent with our observations made in earlier experiments where injection groups had higher mortality compared to non-injection groups (data not shown). In fish that did not receive injections, only one fish in the etomidate group died. Overall, no clear differences emerge on the safety of the anesthetics, based on fish weight and mortality.

Doses of the anesthetics used vary significantly between researchers and laboratories (41). Slow induction of anesthesia based on the anesthetic agent and dose used will increase stress experienced by the fish, making the experienced stress partly proportional to the anesthesia induction time. The anesthesia induction time was similar between the anesthetic agents at all three timepoints (~2.5 min), except at 2. day eugenol provided anesthesia more rapidly than etomidate. A similar anesthesia induction time has been reported with the same tricaine concentration (42), whereas a slightly faster anesthesia induction time (~1.5 min) has been reported with a higher dose of tricaine (400 mg/l) used for euthanasia (45). Measurement of the anesthesia induction time is clearly the most subjective of the parameters evaluated in this study.

The etomidate fish recover slowly from anesthesia (~5.5 min) when compared to both eugenol (~2.5 min) and tricaine (~1.5 min). Similar results indicating slow recovery from anesthesia with etomidate have been shown recently (46). Ethanol was used as a solvent for eugenol and etomidate, resulting in ethanol concentrations of 0.055% and 0.4%, respectively, in the anesthetic solutions used. Ethanol is known to affect zebrafish behavior (47). However, in this study the exposure time to the anesthetic solution was short (~2.5 min). Furthermore, similar concentrations of ethanol for similar exposure times have been shown to increase time spent in the top-part of the novel tank (47). In contrast, we observed that etomidate fish spend less time in the top-part of the novel tank. However, we cannot rule out the potential effect of ethanol on the behavioral results of the eugenol and the etomidate fish. The recovery time from anesthesia is similar for eugenol and tricaine at all time-points. The average recovery times are slightly shorter in the fish that received injections. This is due to these fish being taken out of the anesthetic solution to receive the intraperitoneal injection prior to transfer to the novel tank for recovery. Delayed recovery from anesthesia in zebrafish can have several potential effects, including stress and behavioral changes. A delayed recovery may indicate prolonged stress and lead to abnormal behaviors such as sluggishness, disorientation, or difficulty in coordination (46).

The effect of each anesthetic on recovery from anesthesia is further studied by the time-spent in the top-part of the novel tank and entries to the top-part of the novel tank, which have been shown to associate with reduced anxiety and stress levels (26–29). Ten sham fish were handled without an anesthetic agent. Tricaine fish spend clearly the most time in the top-part of the novel tank, at 2. day even more than the sham fish. It is possible that the anesthetized fish were still partly sedated and therefore less susceptible to stressors in the novel tank. Additionally, perhaps this possible sedation of the anesthetized fish in combination with increased stress caused by handling in the un-anesthetized sham fish caused the observed difference in time spent in the top-part of the novel tank. The difference in time spent in the top-part of the novel tank is clearest between the tricaine fish and the etomidate fish, which spend the least amount of time in the top-part of the novel tank. At 2. day, etomidate fish spend less time in the top-part of the novel tank than sham fish. The differences are the clearest at the beginning of the 10-day protocol. No differences are seen between tricaine fish and eugenol fish, nor between eugenol fish and sham fish. Results are largely similar with and without nociceptive stimuli (injections). The number of entries to the top-part of the novel tank and turnings follow a similar trend, with etomidate fish showing the least entries and turnings. The number of entries and turnings are similar in tricaine, eugenol and sham fish. These findings indicate that eugenol and tricaine seem to associate with the least stress after recovery from anesthesia, whereas etomidate seems to prolong recovery from anesthesia. To confirm our evaluation of when a fish is deemed recovered from anesthesia, the videos were further divided into first half and second half 2.5-min videos and re-analyzed for turning and time spent in the top-part of the novel tank. The tank place preferences and number of turnings remain similar between the two halves in all groups and time-points (Supplementary Figures 1–6, Supplementary figure legends in Supplementary Table 2). Furthermore, since each fish within a group entered the novel tank individually with varying numbers of other group members already in it, we examined whether group size affects swimming behavior in the novel tank. No clear group size effects on swimming behavior were observed.

Acute cortisol levels are low in eugenol fish. Fish quickly metabolize eugenol, making it a safe anesthetic for the food industry (7, 11) and for handling fish (48). Furthermore, eugenol inhibits platelet aggregation and results in higher blood volume collection after euthanasia (49), and may therefore be useful for euthanizing and blood collection at the end of an experiment (49). However, eugenol is a suspected carcinogen and may cause liver problems, possibly limiting its use (35). Furthermore, recent data suggest eugenol might accumulate in the fish after longer exposures, prolonging recovery from anesthesia (42). Etomidate has been shown to be safe to use and to induce deep anesthesia for long durations with good recovery rates (15, 16). Conversely, we found the only fish that died without interventions was in the etomidate group. But these numbers are too small to draw any conclusions. Major drawbacks of etomidate manifest as muscle twitching and involuntary movements during anesthesia, a contraindication to performing procedures, as reported previously (16). The etomidate fish, with and without injections, have the smallest number of entries to top-part, as well as turnings, compared to all other groups at all time-points, clearly demonstrating the prolonged recovery after anesthesia with etomidate.

Acute and chronic cortisol levels are similar with all anesthetics. In the chronic stress measurements, etomidate fish show a trend of lower cortisol levels (Figures 6A, B). Etomidate is known to inhibit cortisol production (3, 14, 34, 50) by blocking adrenocorticotropic hormone stimulation at the inter-renal gland and the mitochondrial cytochrome P450 enzymes, which catalyze cortisol synthesis (34, 50). When cortisol levels are measured acutely after a single stressful event, the etomidate fish show similar levels of cortisol, without a trend of lower cortisol levels (Figure 6C). Acutely the eugenol fish show a trend of lower cortisol levels, approximately half of that seen in all other groups (Figure 5C). To what extent are cortisol levels associated with the amount of stress experienced and whether pharmaceutically lowering cortisol levels decrease the amount of stress experienced, are fascinating questions meriting future studies, but are beyond the scope of this article. It is known that in fish cortisol decreases rapidly after removal of the stressor (51), likely contributing to our finding of lack of cumulative increase in cortisol levels. Future studies may examine how the decreased movement/prolonged recovery from anesthesia and cortisol levels are related in fish anesthetized with etomidate.

Limitations of our study include a low number of fish and possible subjectiveness of the observer when assessing the stages of anesthesia. Although the exposure to the anesthetic agents was repetitive (on 10 days), the single exposure time was brief (only enough to reach stage 4 anesthesia). This may explain why we failed to see significant cumulative effects. We have not tested several anesthetics used in zebrafish, for example lidocaine and benzocaine. In this study the cause of decreased movement after anesthesia, measured as decreased time in the top-part of the novel tank, entries to the top-part, and turnings, cannot be differentiated between increased stress and prolonged recovery from anesthesia. In further studies, measurement of physiological variables, such as heart rate and opercular movement, may give additional valuable information.

In conclusion, our study finds that zebrafish anesthetized with tricaine, and eugenol show fast recovery times. In behavioral studies with the novel tank method (27–31), tricaine fish and eugenol fish show high amounts of turning and the tricaine fish spend the most time in the top-part of the novel tank. Conversely, zebrafish recover slowly from anesthesia with etomidate. Etomidate does not provide analgesia and is known to cause muscle twitching during anesthesia. Our study supports the use of tricaine as the anesthetic of choice for repetitive anesthesia of short duration in zebrafish, followed closely by eugenol, which is a worthy alternative.

## Data Availability

The raw data supporting the conclusions of this article will be made available by the authors, without undue reservation.
