# Supplementary material for: Tricaine, eugenol and etomidate for repetitive procedural anesthesia in adult zebrafish, Danio rerio: effect on stress and behavior
Source: Front Vet Sci. 2025 May 14;12:1562425. doi: 10.3389/fvets.2025.1562425 (PMC12117371; doi:10.3389/fvets.2025.1562425)
Supplement: Supplementary file 1 [file Table_1.docx]

| **Group** | **Average weight (g)** | **n** | **±SEM** |
| --- | --- | --- | --- |
| Tricaine | 0.27 | 5 | 0.02 |
| Eugenol | 0.24 | 5 | 0.02 |
| Etomidate | 0.28 | 5 | 0.03 |
| Sham | 0.25 | 5 | 0.02 |
| Control | 0.23 | 5 | 0.02 |

Supplementary table 1. Weight of fish in acute stress experiment.
