# Supplementary material for: Tricaine, eugenol and etomidate for repetitive procedural anesthesia in adult zebrafish, Danio rerio: effect on stress and behavior
Source: Front Vet Sci. 2025 May 14;12:1562425. doi: 10.3389/fvets.2025.1562425 (PMC12117371; doi:10.3389/fvets.2025.1562425)
Supplement: Supplementary file 2 [file Table_2.docx]

**Supplementary figure legends**

Supplementary Figure 1. Top-part preference comparison of two halves in the novel tank day 2. (%) (A) Tricaine (n=10) (B) Eugenol (n=10) (C) Etomidate (n=10) (D) Sham (n=6) (E) Tricaine (INJ) (n=3) (F) Eugenol (INJ) (n=10) (G) Etomidate (INJ) (n=10).

Supplementary Figure 2. Top-part preference comparison of two halves in the novel tank day 5. /6. (%) (A) Tricaine (n=10) (B) Eugenol (n=9) (C) Etomidate (n=8) (D) Sham (n=6) (E) Tricaine (INJ) (n=3) (F) Eugenol (INJ) (n=10) (G) Etomidate (INJ) (n=10).

Supplementary Figure 3. Top-part preference comparison of two halves in the novel tank day 10. (%) (A) Tricaine (n=10) (B) Eugenol (n=10) (C) Etomidate (n=10) (D) Sham (n=3) (E) Tricaine (INJ) (n=3) (F) Eugenol (INJ) (n=7) (G) Etomidate (INJ) (n=10).

Supplementary Figure 4. Turning comparison of two halves in the novel tank day 2. (n) (A) Tricaine (n=10) (B) Eugenol (n=10) (C) Etomidate (n=10) (D) Sham (n=6) (E) Tricaine (INJ) (n=3) (F) Eugenol (INJ) (n=10) (G) Etomidate (INJ) (n=10).

Supplementary Figure 5. Turning comparison of two halves in the novel tank day 5. /6. (n) (A) Tricaine (n=10) (B) Eugenol (n=9) (C) Etomidate (n=8) (D) Sham (n=6) (E) Tricaine (INJ) (n=3) (F) Eugenol (INJ) (n=10) (G) Etomidate (INJ) (n=10).

Supplementary Figure 6. Turning comparison of two halves in the novel tank day 10. (n) (A) Tricaine (n=10) (B) Eugenol (n=10) (C) Etomidate (n=10) (D) Sham (n=3) (E) Tricaine (INJ) (n=3) (F) Eugenol (INJ) (n=7) (G) Etomidate (INJ) (n=10).
