# Supplementary figures and images for: Tricaine, eugenol and etomidate for repetitive procedural anesthesia in adult zebrafish, Danio rerio: effect on stress and behavior
Source: Front Vet Sci. 2025 May 14;12:1562425. doi: 10.3389/fvets.2025.1562425 (PMC12117371; doi:10.3389/fvets.2025.1562425)

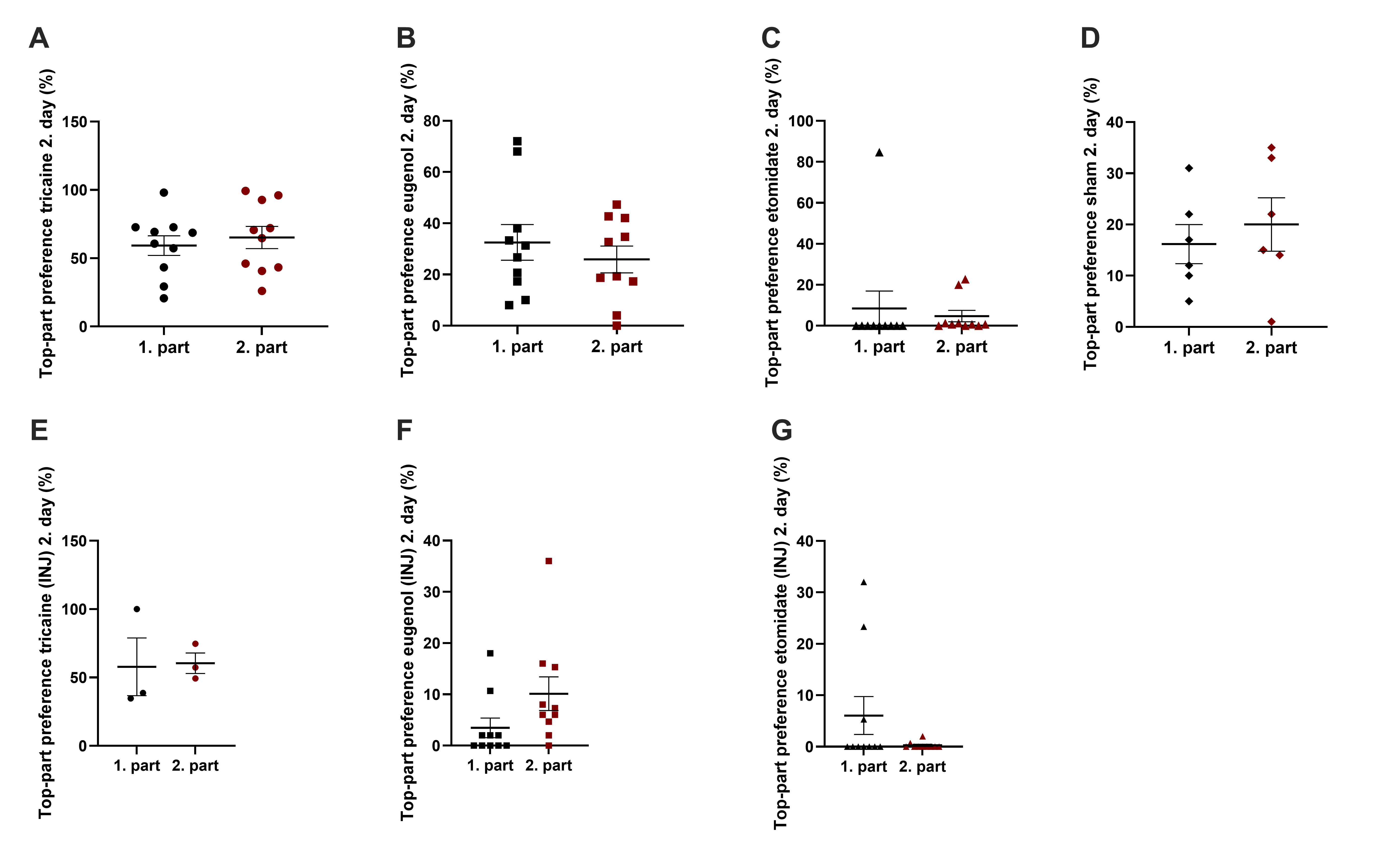

Supplement: Supplementary file 3 [file Image_1.tif]

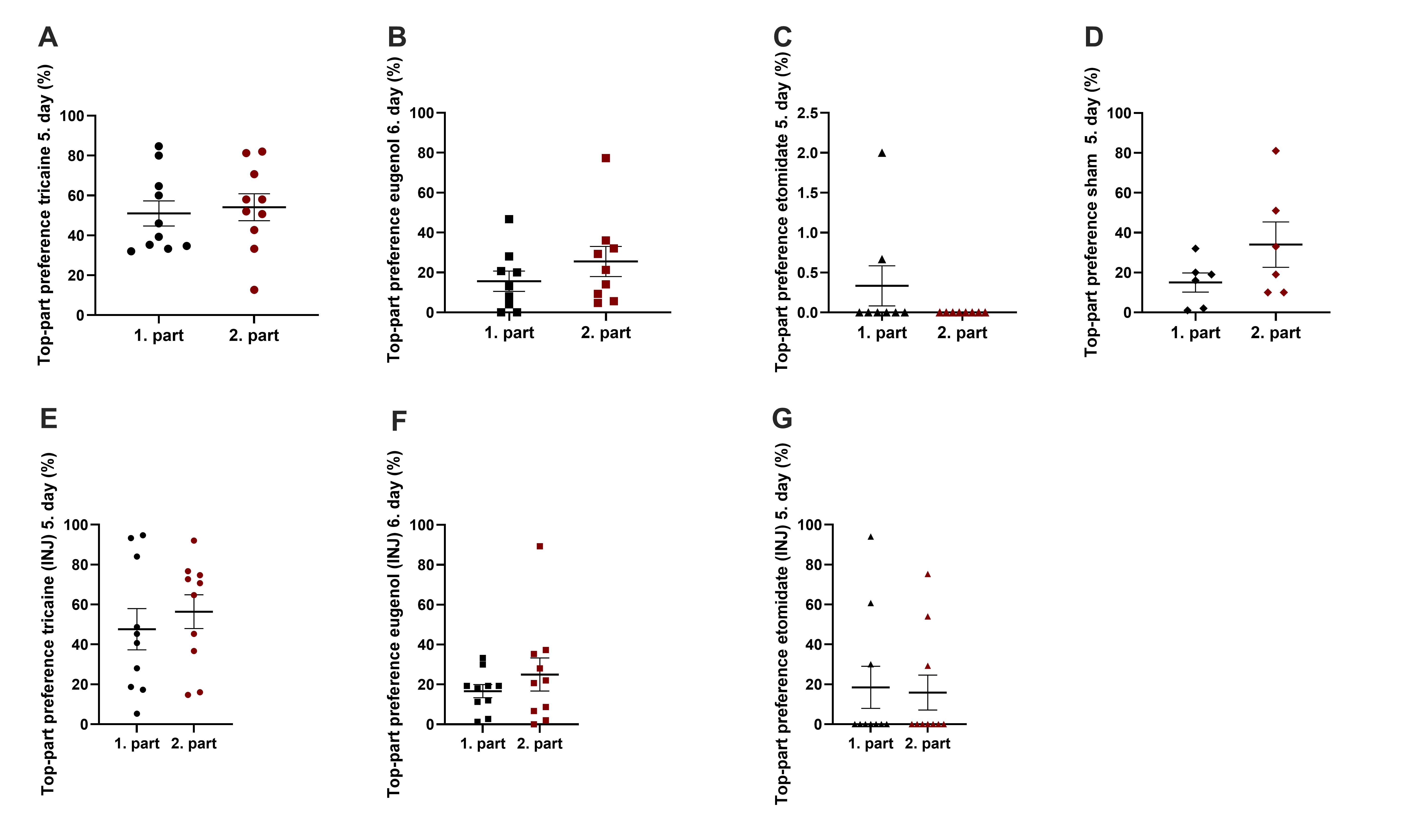

Supplement: Supplementary file 4 [file Image_2.tif]

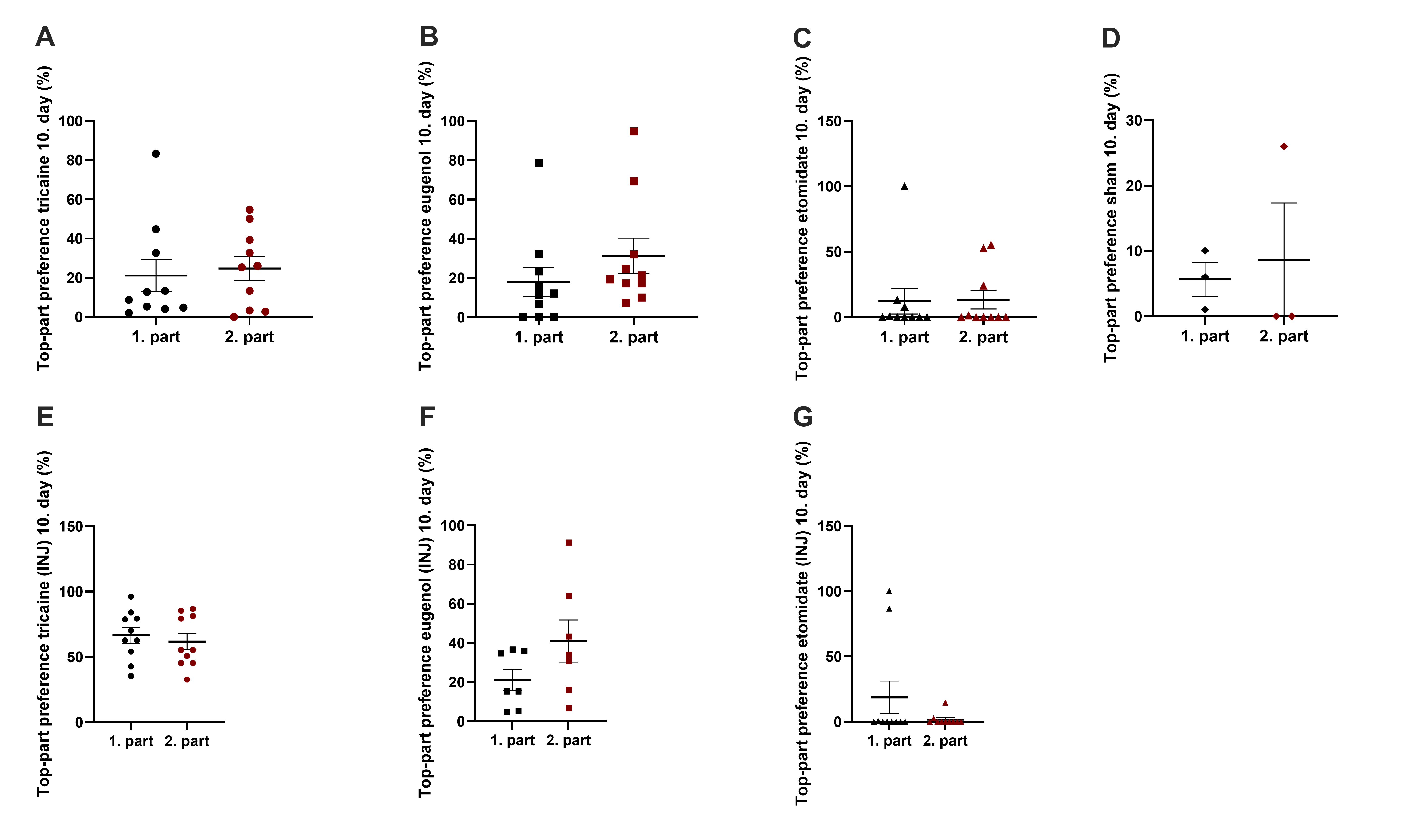

Supplement: Supplementary file 5 [file Image_3.tif]

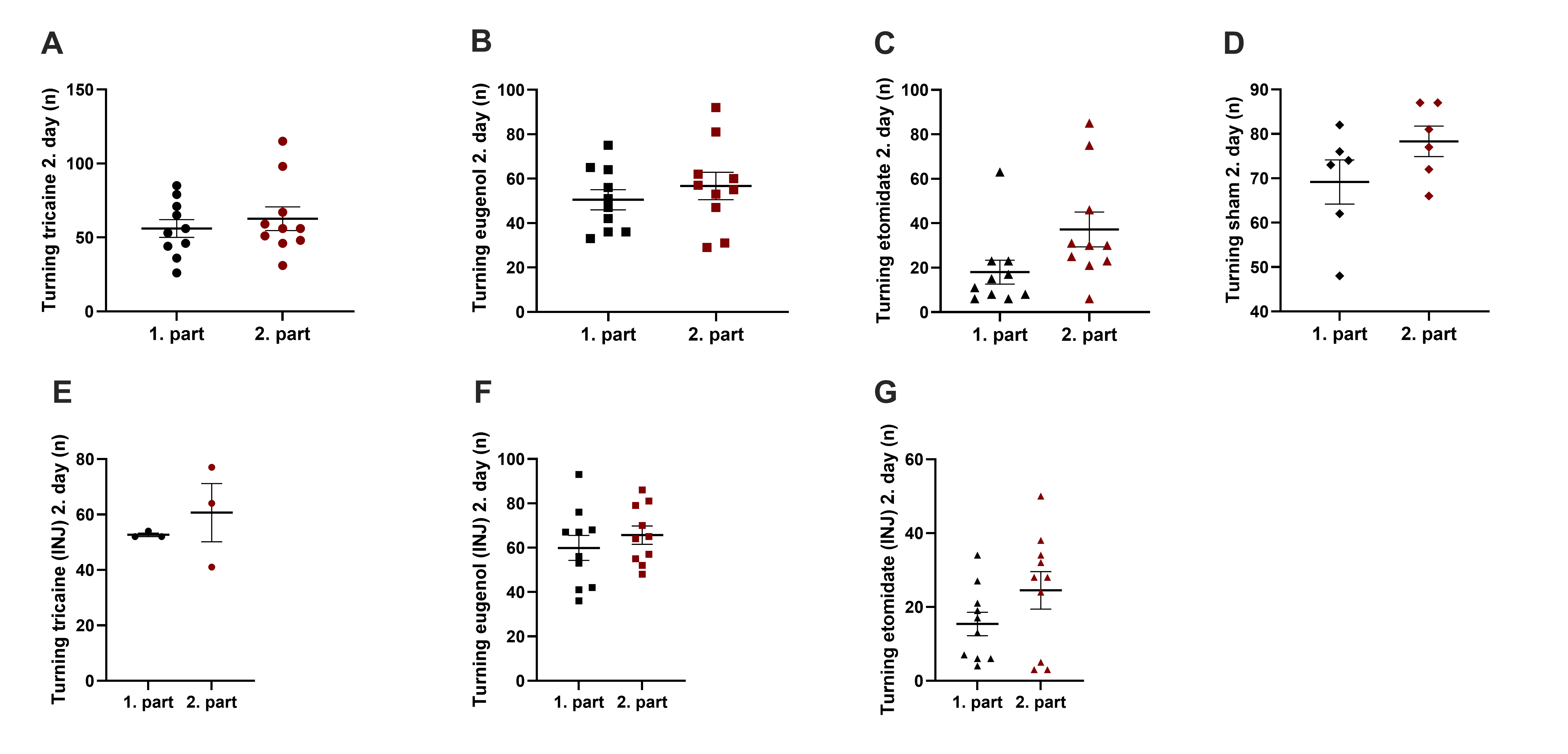

Supplement: Supplementary file 6 [file Image_4.tif]

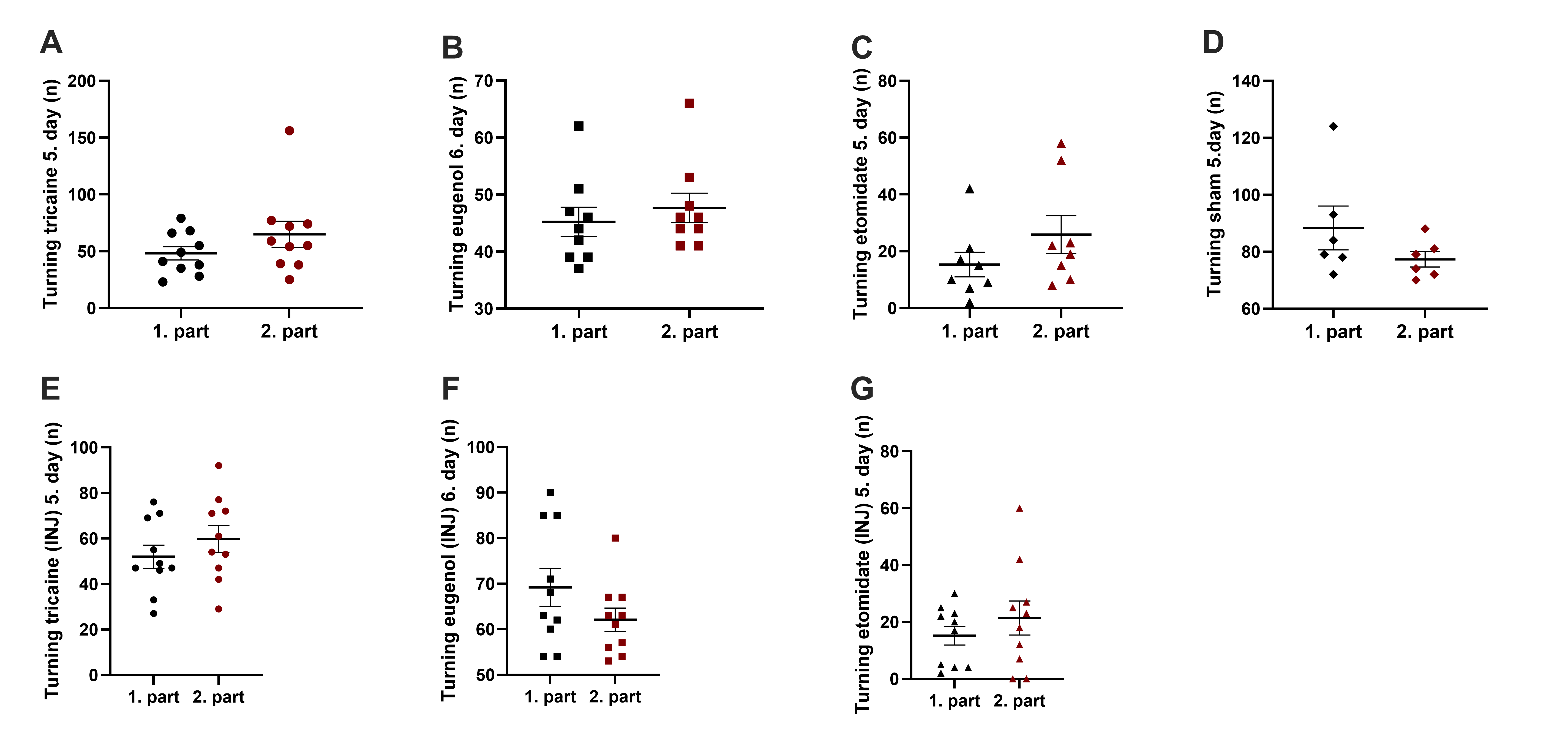

Supplement: Supplementary file 7 [file Image_5.tif]

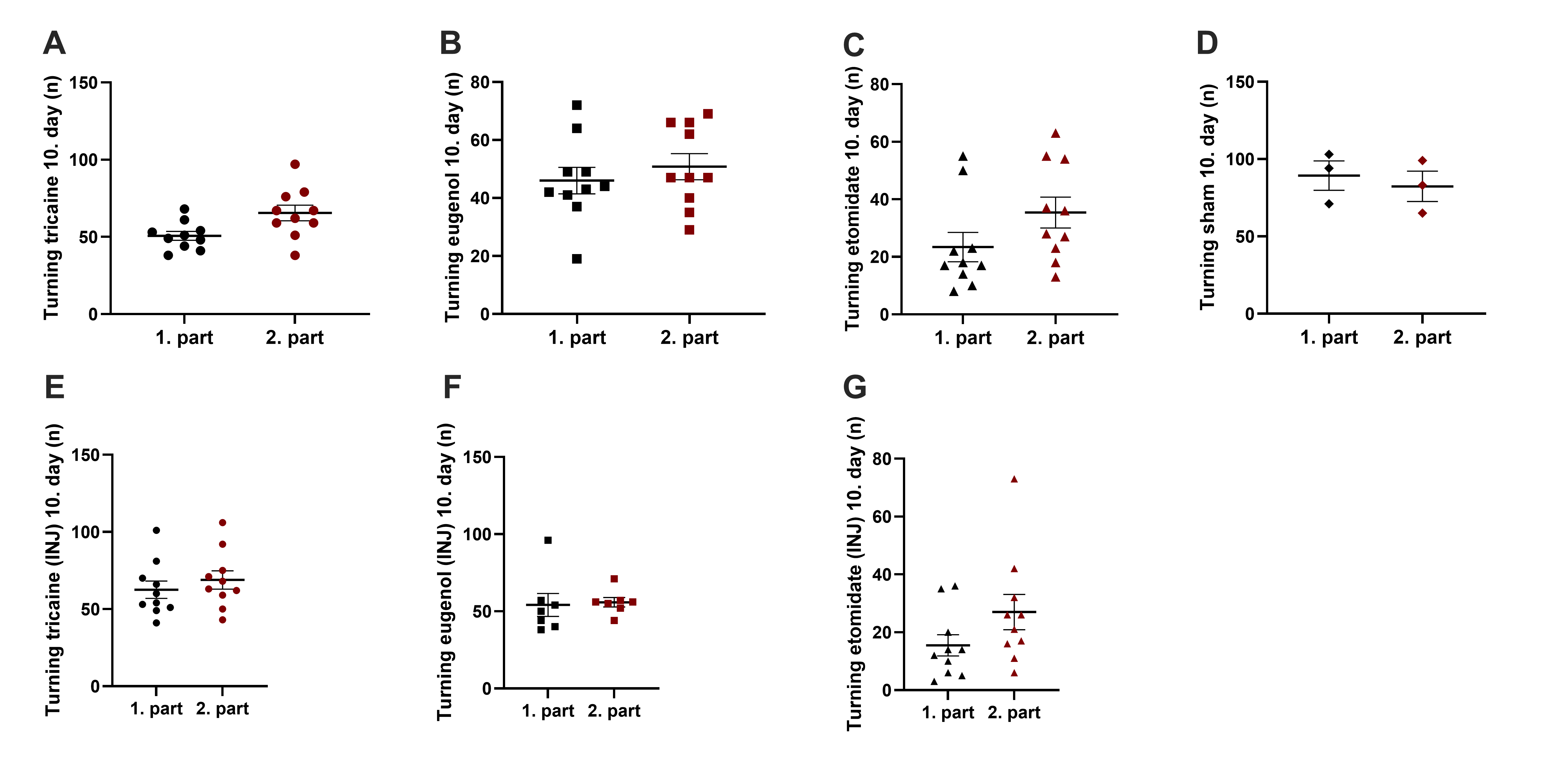

Supplement: Supplementary file 8 [file Image_6.tif]
